# Synthesis of Silver Nanoparticles by Continuous Flow Plasma Discharge with D-Xylose

**DOI:** 10.3390/nano16100631

**Published:** 2026-05-19

**Authors:** Muhammad Aamir Bashir, Ahmad Mukhtar, D. Eric Aston, Sarah Wu

**Affiliations:** Department of Chemical and Biological Engineering, University of Idaho, Moscow, ID 83844, USA; mian.aamir8@gmail.com (M.A.B.); mukh9219@vandals.uidaho.edu (A.M.); aston@uidaho.edu (D.E.A.)

**Keywords:** silver nanoparticle, continuous flow, liquid phase plasma discharge, non-thermal plasma

## Abstract

The scalable production of high-quality nanoparticles is a significant challenge for advancing nanotechnology applications. This research introduces a continuous-flow liquid-plasma discharge reactor for the synthesis of silver nanoparticles at room temperature and atmospheric pressure, utilizing D-xylose as a dual-function reducing and stabilizing agent. The reactor effectively generated uniform xylose-capped silver nanoparticles (X-Ag NPs). Optimal conditions were established utilizing argon gas at a 1:100 molar ratio of Ag precursor to D-xylose, resulting in spherical X-Ag NPs with an average size of 16.89 nm, a zeta potential of −38.87 mV, and a polydispersity index of 0.22. The formation and properties of X-Ag NPs were confirmed through characterization techniques including UV-Vis spectroscopy, dynamic light scattering (DLS), Fourier-transform infrared spectroscopy (FT-IR), and scanning electron microscopy with energy-dispersive X-ray spectroscopy (SEM-EDS). The findings demonstrate that uniform particle nucleation and growth occurred due to the homogeneous distribution of high-energy electrons and reactive gas species produced in the plasma phase. This environmentally sustainable, continuous-flow method shows considerable promise for the industrial-scale production of biomass-derived silver nanoparticles.

## 1. Introduction

Owing to the unique properties of the nano-sized particles compared to their bulk phase, such as their high surface-to-volume ratio, significant research has been carried out over the past few decades [1]. Among different nanoparticles, the silver (Ag) nanoparticles are significantly investigated because of their wide variety of applications, including but not limited to sensing [2], optoelectronics [3], catalysts [4], chemical and biomedical engineering [5]. Different synthesis techniques have been used to control the shape, size, and particle size distribution of the nanoparticles for their targeted oriented applications, and these methods use different reaction conditions to manipulate the nanoparticles’ characteristics [1,6].

Among different methods, the chemical reduction is still considered a practical route, with a drawback of long reaction time, usually several hours, and utilization of toxic reducing agents [7]. As a potential alternative, several researchers devoted their efforts to develop alternative methods using a clean reduction route, which is known as the plasma in or over liquids, where the electrons serve as a reducing agent [8,9,10]. In non-thermal plasma, the applied electric field enables electrons to reach higher temperatures (several eV) to initiate ionization and chemical reactions. At the same time, ions and molecules (i.e., carrier gas) remain at room temperature. Such conditions significantly reduce the reaction time from several hours to seconds without using any additional toxic reducing agents [11].

Among different configurations of the liquid plasma systems, the micro-plasma [9,12], DC glow discharge [13], liquid phase plasma reduction [14], arc discharge [15], and the plasma electrochemistry [16,17] are the most commonly used for the synthesis of Ag nanoparticles. However, most of them are either pin-to-pin and pin-to-plate, which are batch and low in energy efficiency due to the additional energy requirements for gas cavitation during discharge onset, thus pushing towards the development of new configurations [18,19]. This scenario calls for a new configuration that enables continuous, energy-efficient plasma synthesis of nanoparticles in liquids.

Among plasma configurations, the solution plasma process (SPP) and atmospheric pressure plasma jets (APPJs) have been widely used to synthesize Ag nanoparticles. Kondeti et al. [20] demonstrated that the atomic hydrogen radicals driven reduction at the plasma-liquid interface resulted in a narrow size distribution of 2–3 nm of the Ag nanoparticles in a radio frequency (RF) driven atmospheric pressure Ar/H_2_ plasma jet. Habib et al. [1] demonstrated that a tunable particle size distribution can be achieved by manipulating the Ag precursor and capping agent concentrations, leading to the synthesis of Ag nanoparticles within 5 min of plasma irradiation in the He-APPJ. The size of bactericidal Ag nanoparticles was tuned by varying the plasma exposure time to achieve temporal control over nanoparticle growth in a dielectric barrier discharge (DBD) configuration, resulting in particle sizes ranging from 5.4 to 17.8 nm [21]. For in-liquid plasma processes, Lee et al. [22] demonstrated that manipulating gas bubble formation between the silver electrodes and the voltage pulse can control the shape and size of the Ag nanoparticles, resulting in spherical or nanoplate morphologies depending on the discharge conditions. Xu et al. [23] synthesized the size-tunable Ag nanoparticles with a size of 6–20 nm in glycerol as a solvent using a low-pressure inductively coupled plasma (ICP), where the pulsed operation resulted in a significant improvement in mono-dispersion. More recently, Chen et al. [24] reported the rapid synthesis of colloidal nanoparticles of the platinum group metals (PGMs), including Ag, gold (Au), platinum (Pt), and palladium (Pd) in an atmospheric pressure surface DBD using water-ethanol as solvent, where the reactive species, such as the hydrogen radicals and hydrated electrons, drive the reduction process. Collectively, these studies underscore that manipulating plasma operational parameters can help control the shape and size of nanoparticles.

D-xylose has been selected for its dual role of stabilizing and acting as a reducing agent due to its aldopentose nature, which carries a free aldehyde group along with multiple hydroxyl groups, enabling it to perform this dual role towards Ag^+^. It has been established that the reduction of Ag^+^ to Ag^0^ proceeds via the oxidation of aldehydes to aldonic acids. At the same time, the resulting carboxylate and hydroxyl functional groups are adsorbed onto the nascent Ag^0^ nanoparticles, providing electrostatic and steric stabilization against agglomeration [25,26,27]. In subsequent work, it has been confirmed that carbonyl- and hydroxyl-based biomolecules, ranging from monosaccharides to polysaccharides such as cellulose and starch, prevent the agglomeration of Ag nanoparticles via surface coordination at the nanoparticle interfaces and hydrogen bonding [28]. It has also been found that xylose offers additional benefits, including well-defined stoichiometry, higher aqueous solubility, and cleaner surface chemistry for post-synthesis characterization of the nanoparticles, compared to high-molecular-weight polysaccharides, thus making it a promising candidate as an Ag nanoparticle stabilizer in the present system.

In this work, we reported a new plasma reactor configuration for the efficient production of biomass-driven Ag nanoparticles via continuous liquid plasma discharge (CLPD), followed by characterization, stability testing, and a proposed synthesis mechanism.

## 2. Materials and Methods

### 2.1. Chemicals

All the chemicals used in this research, including the deionized (DI) water, D-Xylose, and Silver Nitrate, were purchased from Fisher-Scientific (Waltham, MA, USA). Argon gas (99.99% purity) was purchased from the Chemistry Store of the University of Idaho with maximum purity and used without further purification.

### 2.2. Reactor Setup and Operation

**Figure 1:**
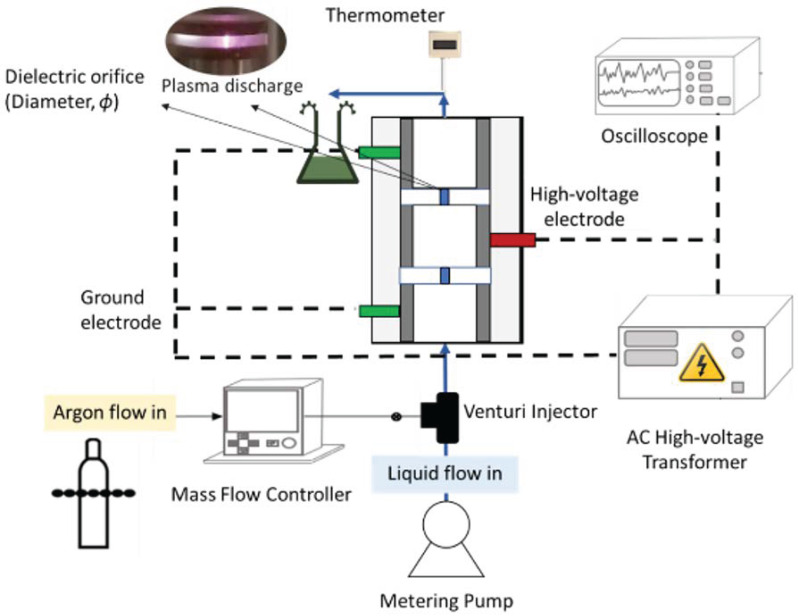
Schematic diagram of continuous liquid phase plasma discharge reactor.

The experimental setup of a two-step continuous liquid-phase plasma discharge reactor (CLPD) is described in Figure 1. The reactor was designed by separating the ground-voltage electrodes from the high-voltage electrodes using dielectric plates with a small opening (1 mm diameter, and 3.24 mm of thickness of each plasma zone) at the center. The discharge was generated in the liquid–gas mixture. The system consisted of a high-voltage transformer (Plasma Technics Inc., Racine, WI, USA) connected to stainless-steel electrodes. A peristaltic pump continuously fed the liquid–gas mixture into the reactor at atmospheric pressure. The transformer regulator could adjust the applied power, and an oscilloscope was used to measure current and voltage. The argon gas cylinder with a gas flow meter was used to add a controlled gas flow to the reactor. The reactor was made of polycarbonate material, and quartz was used for the dielectric plates.

In each experimental setup, 300 mL of aqueous solution containing a different ratio of AgNO_3_:D-xylose with controlled gas was passed (100 mL/min) through the plasma reactor. Once the mixture started to pass through the discharge points, the controlled power was applied (250 W), and the voltage and current in each experimental run were measured using the oscilloscope. After completing the reaction, each sample was centrifuged and washed thrice before resuspending in deionized water for analysis. The solid samples were prepared by drying X-Ag NPs on SEM stubs under vacuum at room temperature for characterization. During the experiment, an oscilloscope (DPO 3034, Tektronix, Beaverton, OR, USA) equipped with a Pearson current monitor (Model 2100) and high-voltage probe (P6015A) was used to capture the current-voltage over time data. Using optical emission spectroscopy (Ocean Optics HDX UV-VIS, Ocean Insight, Orlando, FL, USA) and a fiber-optic probe, plasma-generated species were observed.

### 2.3. Characterizations of Silver Nanoparticles

Each sample was prepared and tested for both the material and optical properties of X-Ag NPs. The ultraviolet-visible light spectroscopy (UV-Vis, Synergy HT, Bio-tek instruments, Winooski, VT, USA) was used for absorbance analysis of X-Ag NPs synthesized in plasma discharge for the maximum absorption wavelength. The dynamic light scattering technique was used for size distribution, zeta potential, and polydispersity index by Brookhaven Instruments ZetaPALS, Nashua, NH, USA. The FTIR analysis was performed using a NicoletTM iSTM 20 FTIR (Thermo Fisher Scientific, Waltham, MA, USA) spectrometer between 650 and 4000 cm^−1^ with a precision of 16 cm^−1^. The FT-IR data was collected using the OMNICTM Specta software (2.1.175). The SEM-EDS analysis was performed using a Zeiss Supra 35 SEM, White Plains, NY, USA. The samples were prepared in a clean environment to prevent sample loss or air contamination.

## 3. Results and Discussion

### 3.1. Characterization of Plasma Discharge

**Figure 2:**
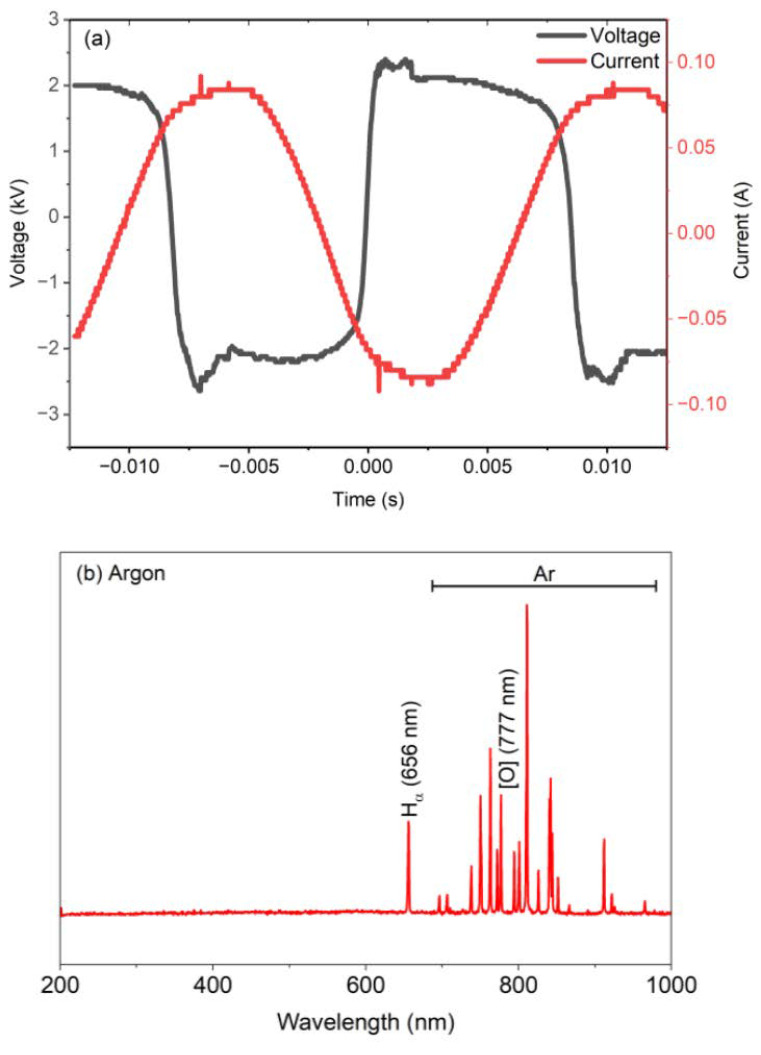
(**a**) Current-voltage behavior over time and (**b**) OES spectra of the plasma-generated species.

The waveform in the voltage-current vs. time graph demonstrated quasi-trapezoidal voltage (~±2 to ±2.5 kV) along with a near-triangular current (~±80 mA) with an applied power of 250 W, collectively indicating predominantly capacitive, a symmetric positive/negative excursion, which further indicates that the plasma discharge is stable with balanced bipolar operation and consistent with the reported AC plasma-liquid and dielectric-barrier discharge (DBD) plasma discharges [29,30,31]. The electric field strength is estimated at 3.9 × 10^5^ V/m at each discharge orifice. The OES spectra (Figure 2) are dominated by the Argon emission lines clustered in the region of 700–850 nm, which arise from the 4p → 4s radiative transitions, with the most prominent line at 811.5 nm, indicating the 2p_9_ → 1s_5_, which overall confirms that Argon has strongly populated excited and metastable states within the discharge [32,33]. The distinct line at 656.3 nm is associated with the Hα Balmer transition, resulting from water vapor dissociation at the plasma-liquid interface, which signifies the active production of reductive species for the Ag^+^ reduction [29].

### 3.2. Functional Groups, Morphology, Elemental and Particle Size Analysis

**Figure 3:**
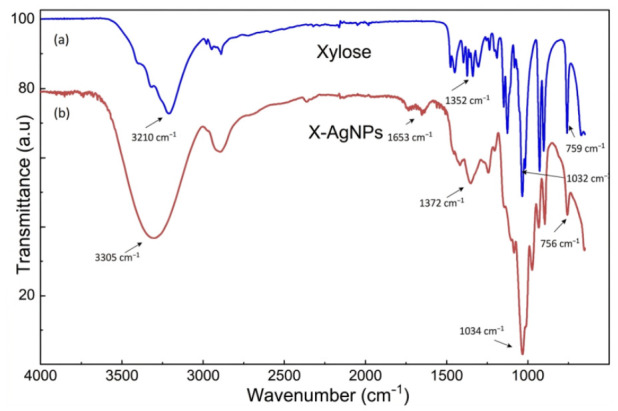
FT-IR spectra of (**a**) the stabilizing agent (xylose) and (**b**) synthesized Ag nanoparticles (X-AgNPs).

To assess the role of D-xylose as a potential capping and stabilizing agent, the presence of the biological functional groups on the synthesized Ag nanoparticles was investigated by using the FT-IR (Figure 3). From the spectra, it is evident that the O-H stretching and deformation peak in xylose at 3210 cm^−1^ made a blue shift to 3305 cm^−1^ in the synthesized Ag nanoparticles, which indicates the successful capping or stabilizing interactions of the xylose with the Ag nanoparticles through the electrostatic interactions [34].

**Figure 4:**
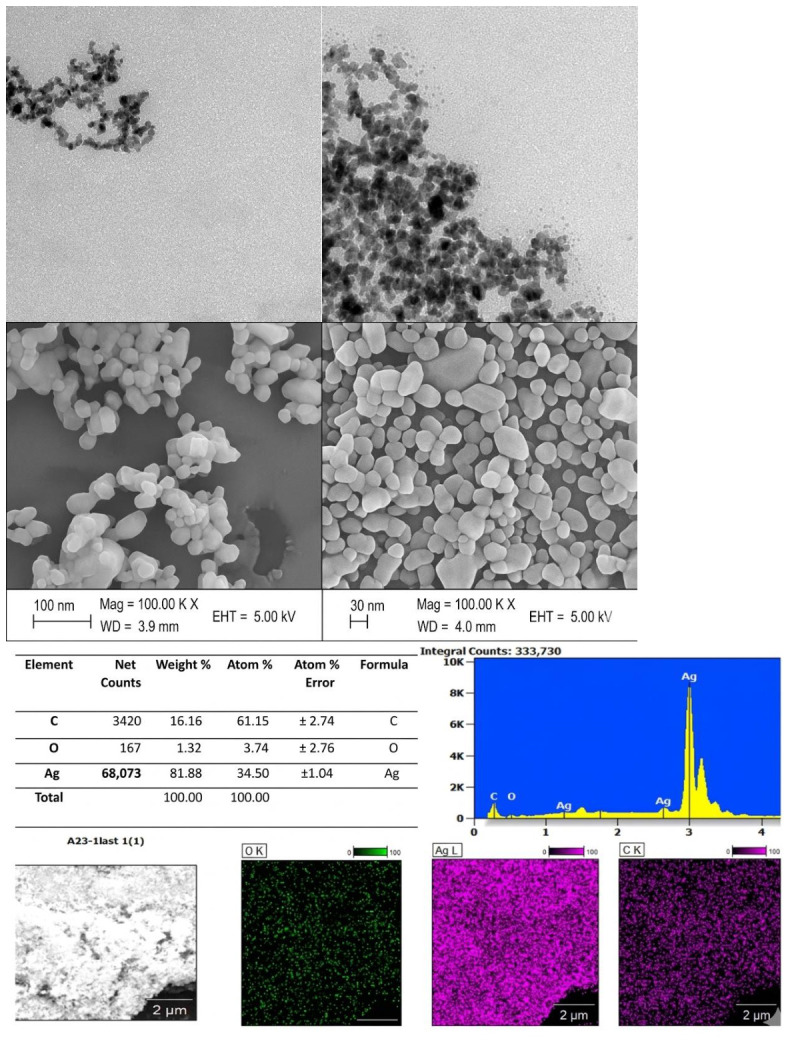
TEM, SEM, EDX, and elemental mapping of the synthesized Ag nanoparticles.

The TEM and SEM images revealed a spherical shape of the synthesized Ag nanoparticles with uneven particle size distribution under all reaction conditions, i.e., different carrier gases. The EDX analysis along with the mapping of the elements revealed that the dominant phase is Ag, which is about 82% (Figure 4).

### 3.3. Influence of Feed Gas, Precursor Concentration, and Input Power

**Figure 5:**
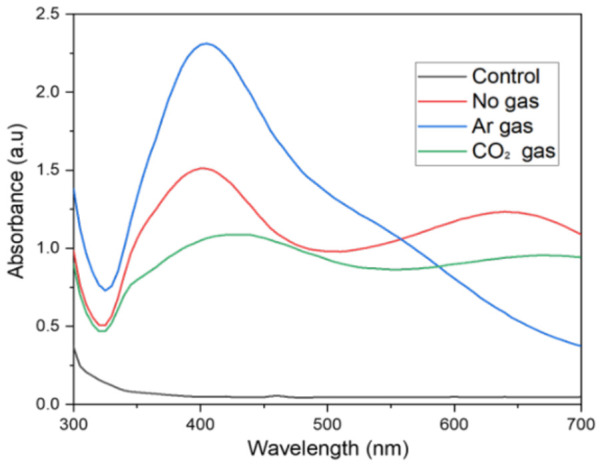
UV-vis spectra of synthesized Ag nanoparticles at different carrier gases.

The UV-Vis spectra illustrate the effects of different plasma gases (no gas, argon, and CO_2_) on the synthesis of silver nanoparticles in the AgNO_3_:D-xylose system (Figure 5). The spectra show variations in peak position, intensity, and broadness, which provide insight into nanoparticle size, distribution, and aggregation. The UV-Vis absorbance spectra indicate significant variations in silver nanoparticle production under varying plasma discharge settings. The control sample exhibits negligible absorbance at all wavelengths, indicating that nanoparticle production requires plasma activation irrespective of gas type. Argon plasma shows a maximum absorbance peak at around 400 nm, indicating optimal silver nanoparticle formation [35]. This observation corroborates the conception that argon’s advantageous ionization characteristics and plasma stability make it highly useful for the synthesis of metal nanoparticles [36]. The “No gas” condition exhibits considerable absorbance at 400 nm, indicating that plasma may still convert silver ions to nanoparticles in vacuum or low-pressure conditions without a carrier gas, but with reduced efficiency compared to argon. Similar research was reported by Marjan et al., where they investigated the plasma reduction of the solid metal precursors in a two-stage process. The first stage involved vacuum generation (200 m-torr), resulting in the complete evaporation of the solvent and leaving behind the solid metal precursor only for the plasma reduction. Such conditions resemble either a low-pressure or no-gas environment while still producing the nanoparticles [35]. It also confirms the unique design feature of the reactor, which can generate stable plasma discharge without carrier gas in liquid flowing conditions, making it distinguishable from conventional pin-to-pin and pin-to-plate configurations. The CO_2_ gas condition exhibits the lowest and broadest absorbance profile, indicating limited nanoparticle formation, which aligns with research showing that CO_2_ plasma is less efficient for direct metal nanoparticle synthesis than argon plasma. The distinctive surface plasmon resonance peak at around 400–420 nm verifies the existence of silver nanoparticles, with peak strength and sharpness directly related to nanoparticle concentration and size uniformity. It is well documented that the CO_2_ plasma discharge dissociates CO_2_ into carbon monoxide (CO) and oxygen species, rather than behaving like an inert gas such as Ar. It has also been demonstrated that the presence of CO_2_ as a carrier gas in a plasma discharge competitively reacts with water molecules, consuming plasma energy and high-energy electrons that would otherwise help reduce metal ions. On the other hand, the formation of oxidative species, such as CO_2_ dissociation products, could also disrupt the reduction of silver ions to metallic silver nanoparticles [37,38]. The enhanced efficacy of argon relative to reactive gases such as CO_2_ is due to argon’s inertness, which inhibits oxidation and facilitates more efficient reduction of silver ions, whereas CO_2_’s reactivity may disrupt the reduction process or promote oxidation, thereby diminishing the overall yield of nanoparticles [20]. Table 1 also presents the average particle size, polydispersity index, and zeta potential in each experiment. The argon gas-induced plasma discharge system generated silver nanoparticles in much smaller sizes mainly because of the high-energy argon radical properties, having different effects compared to CO_2_ and no gas discharge systems, where their significant role in chemical reduction involved electron density generated from electric discharge produced at high power, creating different input energy. In the CO_2_ discharge system, CO_2_ was further reduced into CO and O radicals, allowing a different set of high-energy radicals for interface interaction in liquid and gas compared to the no gas and argon gas discharge systems [24].

**Figure 6:**
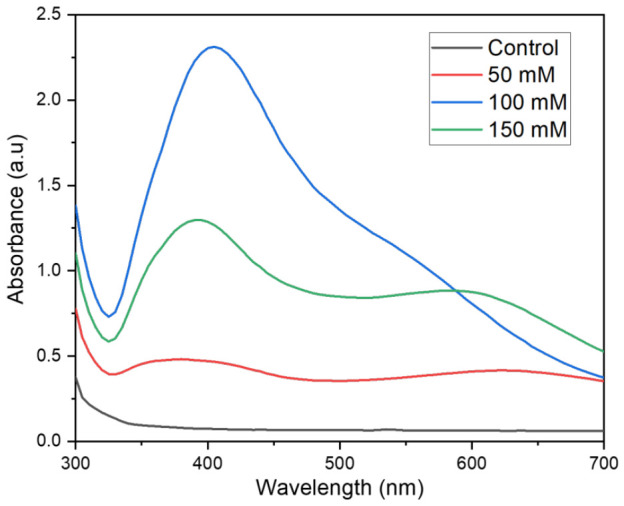
UV-Vis absorption spectra of the Ag NPs synthesized at different ratios of AgNO_3_:D:Xylose under Ar-Plasma.

Figure 6 shows the UV-vis spectra of the silver nanoparticles synthesized at different ratios of AgNO_3_:D:Xylose (control, 50 mM, 100 mM, and 150 mM) under Ar-Plasma. The results revealed that with an increase in silver nitrate concentration, there is a red shift in the peak position, which is consistent with the classical Mie Theory [12]. These results indicate an increase in the synthesized Ag particle size with an increase in the metal precursor. Furthermore, it can also be said that more Ag nanoparticles are produced with increased metal precursor concentration, leading to a red shift in the peak position. As we mentioned, a variation in the metal precursor could lead to a variation in the Ag particle size and aggregation state. AgNO_3_ concentration increases relative to xylose, demonstrating a higher availability of silver ions can lead to the formation of larger nanoparticles. Larger nanoparticles exhibit surface plasmon resonance (SPR) peaks at longer wavelengths, resulting in a red shift in the absorption spectra. Higher silver ion concentrations can also promote nanoparticle aggregation, shifting the SPR peak to longer wavelengths. This phenomenon is consistent with the reported studies where increased precursor concentrations led to red-shifted SPR peaks due to larger nanoparticle sizes and aggregation effects [39]. Corresponding DLS results are tabulated in Table 2, consistent with the UV-vis spectral observations. At a concentration of 50 mM, a strongly non-monotonic dependence of nanoparticle size, dispersity, and surface charge has been observed. At lower concentration, the hydrodynamic size is largest, and the PDI is broadest, indicating that the stabilizing agent was insufficient to sustain the fast nucleation step, which is critical for the narrow size control, which led to the growing and coalescing of the few Ag^0^ nuclei, thus resulting in the larger and polydisperse nanoparticles [40,41]. With an increase in concentration to 100 mM, the size and dispersity showed favorable results, indicating that it successfully established high Ag^0^ supersaturation, driving dense, homogeneous nucleation and rapid passivation of nascent surfaces by xylonic acid-type carboxylate fragments formed by the plasma oxidation of D-xylose [29,42,43]. The corresponding zeta potential also increased to −38.53 ± 5 mV from its conventional threshold value of 30 mV, which is widely accepted as the limit for the electrostatically stable aqueous dispersion with the highest electrophoretic mobility value, thus confirming the robust colloidal stabilization [44]. Further increasing the concentration to 150 mM resulted in large nanoparticle size and a broad PDI, indicating the presence of excessive unreacted D-xylose competing with the adsorbed carboxylate ligand at the Ag surface and partially screening the negative surface charge. It could also be attributed to the accelerated secondary nucleation and Ostwald ripening once the reductant is in stochiometric excess [41,42].

**Figure 7:**
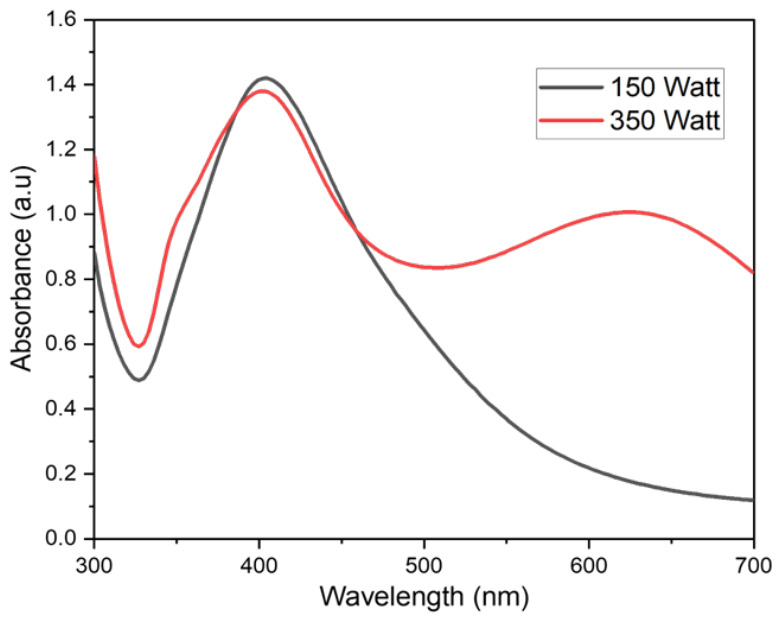
Effect of the applied power on the Ag nanoparticles synthesis.

It has been found that the variation in the applied plasma power significantly influences the UV-vis absorption spectra, particle size distribution, and the surface charge characteristics, probably due to variation in the energy transfer environments under different levels of the applied power (Figure 7). From the results, it can be seen that with an increase in the applied power, the plasmon resonance peak becomes broad with a red shift towards the longer wavelength which indicates an increase in the particle size, resulting in scattering leading to the broadening of the peak and red shift towards longer wavelengths. Dynamic light scattering measurements reveal that increased process time and power broaden the size distribution, with particle sizes ranging from 25 to 130 nm at lower power to 30–350 nm at higher power conditions Table 3. Zeta potential analysis typically shows values around −20 to −22 mV for plasma-synthesized silver nanoparticles, indicating good colloidal stability, though plasma treatment can significantly alter surface charge, changing zeta potential from positive to negative values. Zeta potential values with absolute magnitude greater than 30 mV indicate excellent stability due to strong electrostatic repulsion between particles, while values near zero increase the likelihood of coagulation.

### 3.4. Stability and Mechanistic Insight on Silver Nanoparticle Synthesis

**Figure 8:**
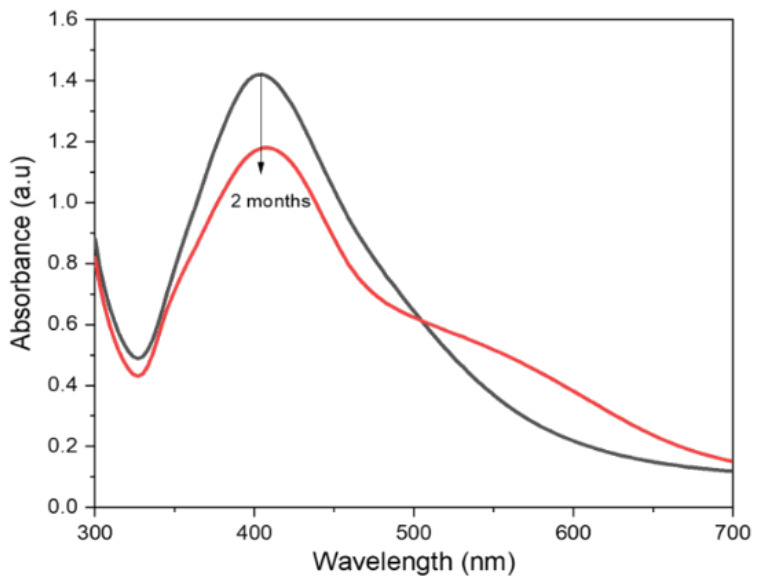
UV-vis spectra of fresh and after 2 months of the synthesized Ag nanoparticles.

It is crucial to establish the stability of the synthesized Ag nanoparticles synthesized via a biomass-based polymer for their potential use in drug delivery systems (Figure 8). To observe the behavior and stability of X-Ag NPs generated in the plasma reactor system, the centrifuged samples were resuspended in PBS media. As shown in Figure 7, a very slight change was observed in nanoparticle dissolution in PBS medium over a 2-month period, which suggested very high stability of X-Ag NPs in biological media, allowing them for long-term use in particularly target-driven chemical reactions in microbial reaction processes.

**Figure 9:**
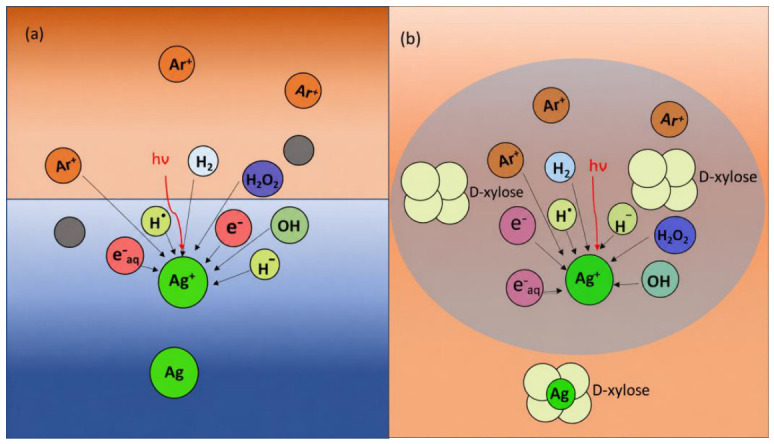
Proposed mechanism of the Ag nanoparticles synthesis (**a**) at liquid surface; (**b**) inside the bubble in continuous liquid plasma discharge.

In this study, metal ions in the liquid are the primary feedstock for the formation of X-Ag NPs in the presence of D-xylose. In most plasma discharge systems, a solid metal electrode provides material in nanoparticle production, such as in the arc discharge method. However, in the liquid phase, nanoparticle production has not been researched at a large scale in labs, and the applications of these nanoparticles have not been fully explored. Nanoparticle formation can be followed through complex physicochemical processes in plasma-liquid reactions because of the presence of the oxidation-reduction species in the plasma system. The plasma contains free electrons and ions with specific energy depending on the discharge mode and free radicals formed by interaction with the liquid plasma discharge. The yield of the reducing and oxidizing species can be adjusted by adjusting the plasma parameters in the plasma phase. The diversity of reducing and oxidizing species associated with liquid plasma discharge complicates the synthesis mechanism analysis but provides new routes for tuning the synthesis process. In addition, plasma parameters can be adjusted by varying the radicals production to control nanoparticles’ shape and size [28]. Figure 9a,b show the processes of the interfacial phases of silver nanoparticles production in plasma at the liquid surface, and on the interface of the current argon gas mixed liquid discharge plasma reactor. In Figure 9a, when a discharge voltage was applied, the high-energy active argon radicals attacked the liquid surface. Depending on applied voltage and gas flow, the argon radicals and electrons provided energy to disrupt the liquid surface and create a large number of sub gases, ions, and free radicals. These secondary effects formed hydrated electrons from water molecules with high reduction potential. Water molecules broke down into OH and H radicals, which was confirmed by optical emission spectroscopy [29,30]. The atomic H radical formed H_2,_ and additional OH and H_2_O_2_ could also be formed, allowing different reducing agents to initiate reduction reactions in other reaction pathways [31]. pH and conductivity of the solution could promote different reducing abilities [31]. There are several interpretations for the synthesis of nanoparticles from liquid discharge plasma. In principle, plasma-generated electrons can reduce metal ions in their metallic forms [32,33,34,35,36]. The reducing agent can be generated automatically during nanoparticle synthesis, which is a significant advantage over conventional solution-based methods. Since the reducing species in the plasma above the surface have limited energy, chemical reactions will occur mainly at the plasma-liquid interface. Figure 9b showed that the plasma formed in the droplets was similar to the plasma above the liquid. However, the active species formed in the bubble dispersed across the plasma-liquid interface, and the reducing efficiency of the liquid was relatively higher than that of the plasma over the liquid [37]. The physical phenomenon of plasma discharge can also add to sputtering and evaporation. When these droplets pass through the plasma phase, depending on the droplet size, the availability of electrons and effect of plasma in the gas–liquid interface can also be changed. To understand the double-layer interface effect, further experiments should also be focused on the droplet size and availability of electrons, radicals, and free ions to effectively produce the uniform-sized metal particles.

## 4. Conclusions

This research demonstrates the effective use of non-thermal liquid-phase plasma technology for the synthesis of uniform biomass-derived silver nanoparticles in a continuous-flow reactor. The gas–liquid mixed plasma system generated X-Ag NPs with radii of 16.89–121.21 nm, depending on the reaction parameters. The key findings indicate that: (1) argon gas plasma discharge yields smaller, more uniform nanoparticles compared to CO_2_ or no-gas systems; (2) optimal production is achieved at a 1:100 AgNO_3_:D-xylose molar ratio with low input power (150 W); (3) D-xylose functions effectively as both a reducing and capping agent, with hydroxyl and carbonyl functional groups being essential for the formation and stability of X-Ag NPs; and (4) the synthesized X-Ag NPs exhibit remarkable stability in phosphate-buffered saline solution over two months, suggesting their potential for biological applications. The uniform energy distribution from the plasma discharge system facilitated homogeneous nucleation, resulting in nanoparticles of consistent size with minimal aggregation. This environmentally friendly, continuous-flow synthesis method offers a viable alternative to traditional batch processes. It demonstrates significant potential for industrial-scale production of silver nanoparticles for antimicrobial, drug-delivery, and biomedical applications.

## Figures and Tables

**Table 1:** DLS measurements of the synthesized nanoparticles at different carrier gases.

Sample	Particle Size (Radius) (nm)	Polydispersity Index (PDI)	Zeta Potential (mV)	Mobility (μm·cm/V·s)
X-Ag NPs (Ar gas)	17.43 ± 5	0.24 ± 0.03	−37.57 ± 5	−2.94 ± 0.03
X-Ag NPs (CO_2_ gas)	32.52 ± 5	0.29 ± 0.03	−35.35 ± 5	−2.69 ± 0.03
X-Ag NPs (No gas)	58.12 ± 5	0.37 ± 0.03	−33.83 ± 5	−2.57 ± 0.03

**Table 2:** DLS measurements of the Ag NPs synthesized at different ratios of AgNO_3_:D:Xylose under Ar-Plasma.

Sample	Particle Size (Radius, nm)	Polydispersity Index (PDI)	Zeta Potential (mV)	Mobility (μm·cm/V·s)
X-Ag NPs (50 mM)	121.21 ± 5	0.41 ± 0.03	−18.57 ± 5	−1.27 ± 0.03
X-Ag NPs (100 mM)	16.89 ± 5	0.22 ± 0.03	−38.53 ± 5	−3.05 ± 0.03
X-Ag NPs (150 mM)	23.32 ± 5	0.29 ± 0.03	−33.83 ± 5	−2.32 ± 0.03

**Table 3:** DLS measurements of the synthesized nanoparticles at different applied powers.

Sample	Particle Size (Radius) (nm)	Polydispersity Index (PDI)	Zeta Potential (mV)	Mobility (μm·cm/V·s)
X-Ag NPs (150 W)	16.43 ± 5	0.25 ± 0.03	−38.87 ± 5	−3.04 ± 0.03
X-Ag NPs (350 W)	44.73 ± 5	0.31 ± 0.03	−35.58 ± 5	−2.78 ± 0.03

## Data Availability

The original contributions presented in this study are included in the article. Further inquiries can be directed to the corresponding author.
